# DeSPPNet: A Multiscale Deep Learning Model for Cardiac Segmentation

**DOI:** 10.3390/diagnostics14242820

**Published:** 2024-12-14

**Authors:** Elizar Elizar, Rusdha Muharar, Mohd Asyraf Zulkifley

**Affiliations:** 1 Department of Electrical, Electronic and Systems Engineering, Faculty of Engineering and Built Environment, Universiti Kebangsaan Malaysia, Bangi 43600, Malaysia; p105681@siswa.ukm.edu.my; 2 Department of Electrical and Computer Engineering, Faculty of Engineering, Universitas Syiah Kuala, Banda Aceh 23111, Indonesia; r.muharar@usk.ac.id

**Keywords:** artificial intelligence, magnetic resonance images, multiscale deep learning, medical image segmentation, semantic segmentation

## Abstract

Background: Cardiac magnetic resonance imaging (MRI) plays a crucial role in monitoring disease progression and evaluating the effectiveness of treatment interventions. Cardiac MRI allows medical practitioners to assess cardiac function accurately by providing comprehensive and quantitative information about the structure and function, hence making it an indispensable tool for monitoring the disease and treatment response. Deep learning-based segmentation enables the precise delineation of cardiac structures including the myocardium, right ventricle, and left ventricle. The accurate segmentation of these structures helps in the diagnosis of heart failure, cardiac functional response to therapies, and understanding the state of the heart functions after treatment. Objectives: The objective of this study is to develop a multiscale deep learning model to segment cardiac organs based on MRI imaging data. Good segmentation performance is difficult to achieve due to the complex nature of the cardiac structure, which includes a variety of chambers, arteries, and tissues. Furthermore, the human heart is also constantly beating, leading to motion artifacts that reduce image clarity and consistency. As a result, a multiscale method is explored to overcome various challenges in segmenting cardiac MRI images. Methods: This paper proposes DeSPPNet, a multiscale-based deep learning network. Its foundation follows encoder–decoder pair architecture that utilizes the Spatial Pyramid Pooling (SPP) layer to improve the performance of cardiac semantic segmentation. The SPP layer is designed to pool features from densely convolutional layers at different scales or sizes, which will be combined to maintain a set of spatial information. By processing features at different spatial resolutions, the multiscale densely connected layer in the form of the Pyramid Pooling Dense Module (PPDM) helps the network to capture both local and global context, preserving finer details of the cardiac structure while also capturing the broader context required to accurately segment larger cardiac structures. The PPDM is incorporated into the deeper layer of the encoder section of the deep learning network to allow it to recognize complex semantic features. Results: An analysis of multiple PPDM placement scenarios and structural variations revealed that the 3-path PPDM, positioned at the encoder layer 5, yielded optimal segmentation performance, achieving dice, intersection over union (IoU), and accuracy scores of 0.859, 0.800, and 0.993, respectively. Conclusions: Different PPDM configurations produce a different effect on the network; as such, a shallower layer placement, like encoder layer 4, retains more spatial data that need more parallel paths to gather the optimal set of multiscale features. In contrast, deeper layers contain more informative features but at a lower spatial resolution, which reduces the number of parallel paths required to provide optimal multiscale context.

## 1. Introduction

Heart disease conditions such as cardiomyopathies, heart failure, and coronary artery disease are the most common causes of mortality among adults in the majority of developed countries and a significant number of developing countries. Heart diseases significantly influence the increase in healthcare expenses that result in significant disability and productivity losses, particularly among the elderly [1]. Accurate diagnosis and early intervention are crucial to reduce the mortality rate and improve treatment outcomes. By identifying risk factors and implementing preventive measures such as lifestyle modifications and medication, healthcare providers can significantly improve treatment outcomes and reduce healthcare costs [2]. Cardiovascular magnetic resonance imaging (cardiac MRI) has emerged as a valuable tool in assessing the disease accurately. By providing detailed images of the heart’s structure and function, cardiac MRI enables clinicians to diagnose heart diseases early, assess disease severity, and monitor treatment response. This information is crucial for selecting appropriate therapies to optimize the patient’s treatment outcomes [3,4]. Cardiac MRI provides details on the left ventricle cavity (LV), right ventricle cavity (RV), and myocardium (Myo), giving insights into the size, shape, function, and tissue characteristics of the heart, which are essential for diagnosing various cardiac conditions such as heart failure, myocardial infarction, and cardiomyopathies. By utilizing advanced imaging sequences, cardiac MRI can provide detailed information about myocardial tissue characteristics, blood flow, and cardiac chamber dimensions [5].

The main advantages of cardiac MRI compared to the other imaging modalities are due to its unmatched soft tissue contrast, non-ionizing radiation, and advanced functional imaging capabilities. Cardiac MRI excels in visualizing myocardial tissue properties, enabling detailed assessments of fibrosis, edema, and scar tissues without the need for ionizing-based imaging such as CT scans. It is particularly crucial in the case of pediatric and pregnant patients, where radiation exposure might bring harmful consequences. Additionally, cardiac MRI provides high-resolution imaging that is less affected by patient conditions and surrounding factors like obesity and poor acoustic windows, which can limit echocardiography’s effectiveness. These advantages make it a reliable tool for screening and diagnosing a wide range of cardiovascular conditions. Cardiac MRI’s reproducibility and objectivity in quantitative measurements (e.g., ventricular volumes and ejection fraction) surpass the variability inherent in echocardiography. Unlike nuclear imaging, which offers metabolic insights but suffers from low spatial resolution and radiation risks, cardiac MRI delivers comprehensive and safe imaging, supporting its role as the gold standard in cardiovascular diagnostics and research.

Cardiac MRI also offers a comprehensive tool for monitoring cardiac disease progression and treatment response. It also provides precise and reliable information about the structural damage associated with cardiomyopathy, both ischemic and non-ischemic [6]. Its non-invasive nature, high spatial resolution, and ability to assess myocardial perfusion, viability, and functional parameters contribute to its role in facilitating clinical decision-making and improving patient care. Moreover, the non-invasive nature of cardiac MRI reduces the risk associated with repetitive imaging, making it a safe and reliable tool for the long-term monitoring of disease progression and evaluating the effectiveness of treatment interventions. Its ability to track changes in cardiac function and tissue characteristics over time enables clinicians to assess the response to pharmacological therapies, cardiac rehabilitation, or surgical interventions. This periodic monitoring helps optimize patient management and tailor treatment strategies to each individual’s needs [7].

In recent years, significant strides have been witnessed in the field of deep learning, particularly in the domain of medical image analysis. One notable advancement is the development of neural network-based automatic semantic segmentation techniques, which have revolutionized the way medical images are analyzed automatically. The semantic segmentation task focuses on labeling each pixel of an image with its corresponding anatomical structures, such as the lungs, optic disc, ligaments, heart, and blood vessels [8,9,10,11]. One of the early cornerstones of architecture in this field is the Fully Convolutional Network (FCN) [12]. The FCN employs a unique architecture that consists of two primary components: a symmetric encoder–decoder pair. The encoder pathway is responsible for extracting relevant features from the input image. This is achieved through a series of convolutional layers that progressively reduce the spatial dimensions of the image while increasing the complexity of the extracted features. Subsequently, the decoder pathway upsamples these features, gradually reconstructing the original image resolution. By combining low-level details with high-level semantic information, the FCN produces accurate pixel-wise segmentation maps.

In the context of heart disease diagnosis using cardiac MRI, the FCN’s ability to process images of arbitrary size plays a transformative role by automating the delineation of key cardiac structures like the LV, RV, and myocardium [13,14]. When it is used in cardiac MRI imaging, the FCN has demonstrated excellent segmentation performance [15,16]. By segmenting cardiac structures, this deep learning model can highlight abnormalities in shape, size, or texture, which may be early indicators of heart disease.

Building upon the foundational work of FCNs, Ronneberger et al. [17] introduced the U-Net architecture, a novel network design that has significantly advanced the field of medical image segmentation. The U-Net’s distinctive feature lies in its use of skip connections, which facilitate the integration of low-level feature details from the encoder path with high-level semantic information from the decoder path. This architectural innovation enables the network to capture both fine-grained details and global context, leading to improved segmentation accuracy, particularly in scenarios with limited training data.

While U-Net has been a milestone in medical image segmentation, recent advances have focused on enhancing feature reuse and gradient flow within the network. In [18], dense CNN architecture was proposed with a promising approach, particularly for medical segmentation tasks. Its unique design has been used in [19], in combination with the FCN model to tackle the challenge of acute ischemic stroke segmentation of diffusion-weighted images (DWIs). This model integrates dense connections to enable each layer to access the feature maps of preceding layers, promoting better information flow and efficient parameter usage. By incorporating densely connected paths, it can achieve a high degree of feature reuse, allowing the model to learn richer representations that capture subtle details essential for precise ischemic stroke segmentation.

Cardiac MRI images consist of a variety of cardiac chamber structures in shape and size. The receptive field of a single-image scale or small convolution kernel is relatively fixed, which means that it is only effective in representing the image features within a specific scope of receptive fields. Consequently, it is unable to accurately depict the edges of various sizes of cardiac chamber structures. Multiscale deep learning techniques can help overcome the challenges in segmenting cardiac MRI images by leveraging information at different spatial resolutions and scales, allowing the segmentation algorithm to understand the overall structure of the cardiac structure while also focusing on finer details. Although multiscale deep learning techniques can significantly improve segmentation accuracy, they often come at the cost of increased computational demands and model complexity. Multiscale deep learning models frequently involve multiple parallel network paths to represent various scale information that increases the computational requirements, which requires the use of high-performance hardware. Designing an effective multiscale deep learning model also requires the careful fine-tuning of hyperparameters and submodule components. This complexity can lead to longer development times and increased difficulty in identifying the optimal configuration.

This paper proposes a novel multiscale deep learning model by utilizing a multiscale and densely connected convolutional network for accurate automatic segmentation of cardiac MRI images. The proposed model, namely, DeSPPNet, utilizes the spatial pyramid pooling (SPP) structure introduced in [20] to capture various spatial resolutions and scales from densely connected convolutional blocks.

The main contributions of this work are as follows:

1. A novel DeSPPNet based on a fully convolutional and multiscale densely connected convolutional network for the automatic segmentation of cardiac MRI.
2. An extensive evaluation of DeSPPNet on the ACDC cardiac MRI dataset with various SPP structures and multiscale dense block placement locations to investigate the generalization capability of the model.
3. A comparative performance evaluation of the SPP multiscale structure with other multiscale structures, namely, ASPP, WSPP, and WASPP.
4. A comparative evaluation of DeSPPNet with state-of-the-art semantic segmentation networks.

This paper comprises five primary sections. The next section discusses the structure of the proposed DeSPPNet. Section 3 provides detailed explanations of the datasets used in this work and the experimental setups. Section 4 discusses the results, while Section 5 provides a concise conclusion.

## 2. DeSPPNet

### 2.1. Spatial Pyramid Pooling (SPP)

Pyramid pooling modules like Spatial Pyramid Pooling (SPP) can aggregate context information at multiple scales, enhancing the network’s capability to learn both fine details and global context. SPP allows the network to effectively segment both small structures like thin myocardium and larger structures like the left ventricle. The SPP layer is designed to pool features from parallel branches at different scales, which will later be combined to maintain spatial information. The primary purpose of SPP is to handle inputs of varying sizes and to create fixed-size outputs that can be processed by fully connected layers regardless of the input image dimensions.

**Figure 1:**
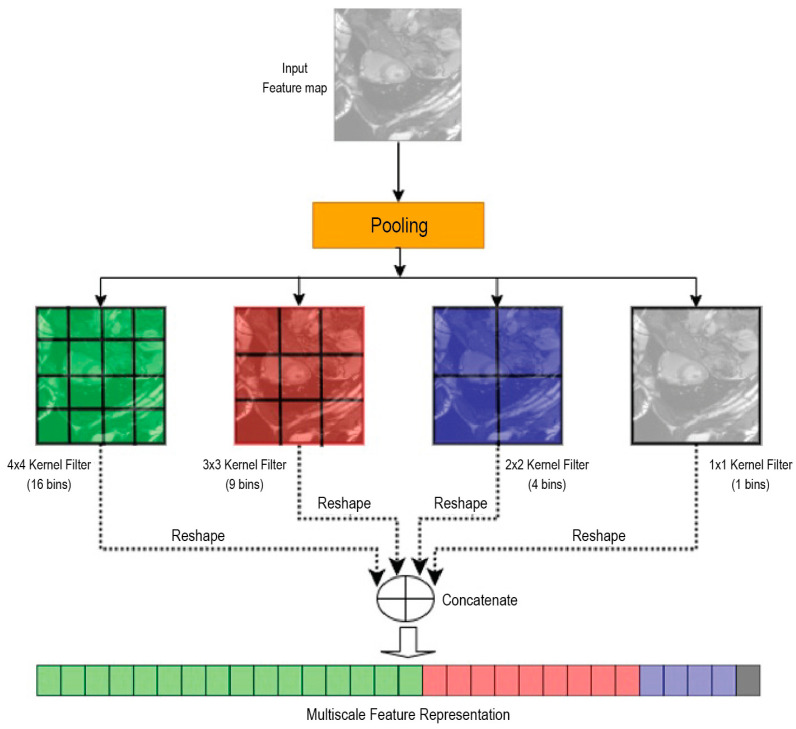
SPP structure with pooling kernel sizes of (1 × 1), (2 × 2), (3 × 3), and (4 × 4).

In an SPP layer, as shown in Figure 1, features are pooled in local pooling kernel across the entire feature map, and these kernels have sizes that correspond to different subdivisions of the feature map (e.g., 1 × 1, 2 × 2, 3 × 3, 4 × 4) [21,22]. As a result, features are captured at multiple scales and encoded into a fixed-length representation. This allows the CNN model to recognize patterns that are spatially invariant to scale, improving its robustness to the size and aspect ratio variations.

The SPP module is always composed of multiple parallel paths, and each parallel path will apply a different pooling kernel size. The pooling kernel plays a critical role in SPP by controlling the size of the resultant feature map for each respective SPP parallel path. A smaller pooling kernel will result in a larger feature map size, while a larger pooling kernel will result in a smaller feature map size. However, each feature map, regardless of its size, contains the same type of features at different scales. A convolution operator (with a fixed convolutional kernel filter size of 3 × 3) will then follow each of the parallel paths to extract the multiscale features of anatomical structures in cardiac MRI. A small feature map size will let a convolutional filter size cover a bigger scale of feature maps; conversely, with the same convolutional filter size, a bigger feature map size will let a convolutional filter capture different scales of features. This multiscale approach enables the model to preserve spatial information across varying resolutions, which is essential for tasks like cardiac MRI segmentation, where both localized anatomical structures and overall organ geometry are important. A balanced combination of different pooling kernel sizes allows the model to build a multiscale representation, integrating detailed and contextual features, enhancing segmentation accuracy and robustness to variations in input image sizes. By adapting the pooling strategy to leverage multiple kernels, SPP ensures that the multiscale deep learning model achieves a balanced representation of features critical for precise and context-aware segmentation.

SPP improves the network’s ability in image recognition tasks by aggregating features from different regions and scales, making the network more effective at capturing global and contextual information while keeping object distortion to a minimum. This makes the network better at extracting comprehensive feature representations and can contribute to improved performance in tasks such as image classification, object detection, and semantic segmentation [23,24].

SPP works as described below:

- Pooling bin SPP divides the feature maps produced by previous convolutional layers into a set of bins at various scales. Each scale corresponds to a level in the pyramid, with the number of bins per level continually increasing. For instance, you might find levels with 1 × 1, 2 × 2, 4 × 4, etc., bins [22].
- There are variable input sizes, whereby a standard CNN model requires a fixed input size to ensure the output volume is consistent for the fully connected layers. SPP allows the network to take images of various sizes and aspect ratios without the need to resize them to a fixed dimension. After the convolutional layers process the variable-sized images, the SPP layer pools the feature maps and produces fixed-length outputs [25].
- Feature concatenation allows a set of features to be extracted from each pyramid level that is pooled (usually by max pooling) and concatenated into a single vector. This vector encapsulates a rich and multiscale representation of the input image’s features.
- There is robustness to object variations due to the model’s capability in capturing features at various levels in the pyramid. The SPP layer then provides robustness against changes in object scales, orientations, and translations since any change in the object’s spatial size causes the feature representation across different pyramid levels to remain effective at capturing the object’s information.

Cardiac MRI images often contain cardiac structures that have variations in size and shape. Therefore, multiscale information is important to capture features of objects with varying sizes, such as ventricles, atria, or small blood vessels. SPP is an advanced multiscale feature extraction methodology that allows the network to capture features from multiple resolutions without requiring changes in input size [26]. By embedding the SPP and dense blocks to the base model, the segmentation network will be better at combining data from different levels of space. The representation will be richer and more accurate, which will aid in producing a more accurate cardiac MRI segmentation.

### 2.2. DeSPPNet Network Structure

**Figure 2:**
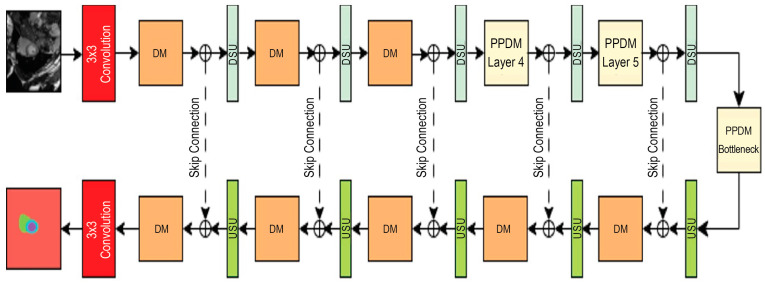
DeSPPNet architecture.

**Figure 3:**
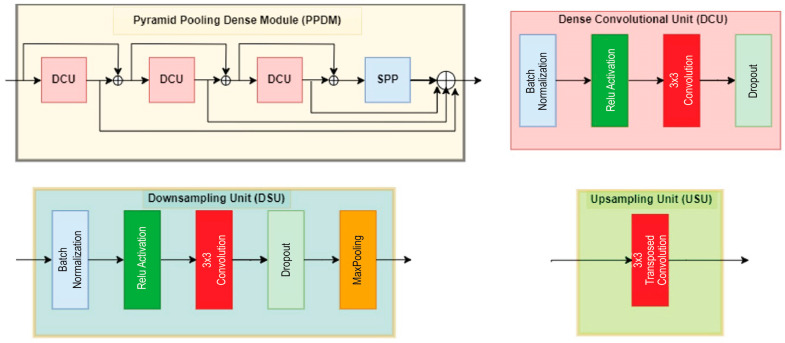
PPDM, DCU, DSU, and USU structures.

Figure 2 shows the DeSPPNet architecture in detail, where the backbone of the DeSPPNet consists of standard multiple layers of CNN, making it ideal for dense predictions where spatially accurate predictions are needed. A standard semantic segmentation network consists of convolutional layers in the encoder and decoder paths, allowing the model to capture both local and global contextual features. In this paper, DM stands for Dense Module, PPDM stands for Pyramid Pooling Dense Module, DSU stands for Downsampling Module, and USU stands for Upsampling Module. The proposed DeSPPNet architecture utilizes several DMs and PPDMs, especially in the encoder path section, to perform feature extraction from cardiac MRI input. Each of these modules accumulates features from its preceding layers, which increases the total number of feature channels. This growth in the channel number is beneficial for capturing rich feature representations, but it can also lead to an overwhelming amount of information, consuming more memory and computational resources. Therefore, in order to optimize the number of feature channels and regulate the complexity of the model while maintaining manageable memory and processing performance, a DSU module is required for each application of a DM. The DM uses skip connections to concatenate feature maps from previous layers, resulting in a more comprehensive feature set. However, the DSU helps regulate this growth and prevents the feature maps from becoming too overly complex, which increases computational resources significantly. The structure of the DSU is shown in Figure 3, which has a bottleneck-like structure, taking out the unnecessary or noisy parts of the cardiac MRI data and leaving only the most important ones. This structure can improve the generalization quality of feature maps by focusing on the most informative aspects of the network.

The PPDM is constructed by integrating a dense structure with the SPP module, which enables it to combine features across scales to improve the segmentation performance in detecting both small and large structures in cardiac MRI data. The PPDM directly connects each layer to its previous paths, facilitating a denser information flow that can be beneficial in medical image segmentation, where fine details and multiscale characteristics are critical for identifying the borders of cardiac anatomical structures [27]. Compared to PPDM, the DM differs slightly in design, whereby it only employs a dense structure (in the form of the same dense convolutional unit (DCU) as the PPDM) to preserve low-level spatial features and high-level contextual features across the network. In cardiac segmentation using MRI, spatial and structural information from the early layers remains relevant for subsequent layers. However, the use of a dense block structure focuses more on direct inter-layer connections and repeated feature collection, which is less optimal for capturing large-scale information. Thus, the application of PPDMs at deeper layers in the DeSPPNet network can help in distinguishing similar cardiac structures, such as different parts of the heart that have different sizes but similar shapes and textures. Reusing features from previous layers in the PPDM enables the network to learn richer feature representations, which is critical for distinguishing subtle variations in cardiac tissues.

The architecture contains the encoder path that aims to capture context and the decoder path that aims to produce precise localization. These paths are connected by the skip connections from the downsampling to the upsampling path to help in the recovery of spatial information lost during downsampling. Then, DeSPPNet uses bottleneck layers to reduce the number of features and compress the information before it is passed to subsequent layers, improving computational efficiency. The decoder path is designed to progressively reconstruct the spatial resolution of the segmented output. The DM and USU layers play important roles in this process. The USU (as shown in Figure 3) module is used to upsample feature maps using transposed convolutions to enable the output to progressively recover the original spatial dimension of the input image. The DM’s role is to refine and integrate feature information using dense connectivity to progressively build up detailed feature maps from lower-resolution feature maps. Unlike the encoder where dense connections capture and compress the context, the decoder focuses on recovering spatial details and precise boundaries of segmented regions. Dense connections in DMs help the network retain both high-level semantic information from deeper layers and local spatial details, which is essential for reconstructing accurate segmentation maps with sharp boundaries. In segmentation tasks, using DMs in the decoder helps prevent loss of detail during upsampling and ensures that fine-grained information is retained across layers, which is critical for tasks like cardiac MRI segmentation where the boundaries between anatomical regions must be precise.

## 3. Materials and Methods

### 3.1. Cardiac MRI Dataset

This study utilized the Automated Cardiac Diagnosis Challenge (ACDC) datasets to fit the deep learning models in order to automate the process of cardiac segmentation, so that cardiac MRI interpretation’s accuracy can be improved. The ACDC dataset was collected from clinical exams acquired at the University Hospital of Dijon (Dijon, France). It includes cardiac MRI scans from 100 patients, divided into five evenly distributed diagnostic categories: healthy subjects, myocardial infarction, dilated cardiomyopathy, hypertrophic cardiomyopathy, and abnormal right ventricle. This stratification ensures a balanced dataset that reflects a wide range of cardiac conditions, hence improving the generalizability of the models trained on it. The addition of both pathological and normal cases supports comprehensive algorithm evaluation across varying anatomical and pathological presentations [4].

Patient selection was designed to maximize diversity in terms of cardiac function, geometry, and pathology while maintaining high data quality. The MRI scans are anonymized, with a consistent imaging protocol including end-diastolic and end-systolic frames for both short-axis views. The dataset also includes expert-annotated ground truth segmentations for the left ventricle, right ventricle, and myocardium, ensuring robust evaluation [4]. These annotated datasets provide a valuable benchmark for validating the clinical relevancy of the developed cardiac-related automation tools. The ACDC dataset consists of secondary data that can be used for research in a controlled setting without needing direct ethical approvals for patient interaction. This makes it a useful tool for improving automated cardiac MRI analysis.

The ACDC cardiac MRI datasets have several key features [28]:

- Diverse imaging scenarios—ACDC datasets reflect real-world clinical scenarios, including images from patients across a wide range of ages, body masses, and cardiac pathologies.
- Standardized imaging protocols—the cardiac MRI data in ACDC datasets were acquired using standardized imaging protocols, making it easier for researchers to compare the performance of their algorithms consistently.
- Volume and timing variability—the datasets include images of various phases of the cardiac cycle, capturing both diastolic and systolic states, which is crucial in modeling the dynamic nature of cardiac function.
- Multimodal data—the dataset provides additional data types like patient demographics and clinical parameters, which can be used to improve the diagnostic capabilities of AI-driven systems.
- Shared annotation guidelines—the ground truth annotations provided with the ACDC dataset follow a certain consensus and protocol, ensuring a high-quality labeling process. The examples of both the images and their respective ground truths are shown in Figure 3.

**Figure 4:**
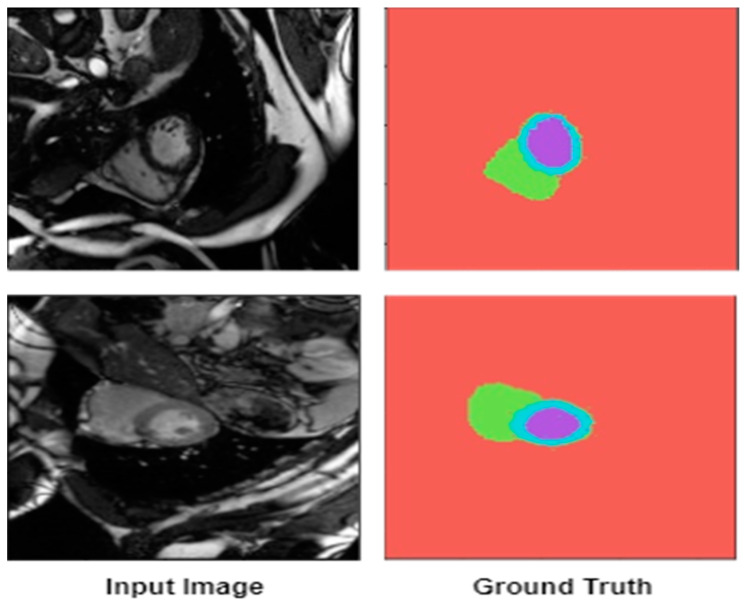
Sample of cardiac magnetic resonance images from the ACDC dataset.

The ACDC cardiac MRI dataset consists of short axis (SA) cine MRI images of 150 patients clinically diagnosed into five classes: 30 cases of normal patients, 30 cases of dilated cardiomyopathy (DCM) patients, 30 cases of hypertrophic cardiomyopathy (HCM) patients, 30 cases of heart failure with myocardial infarction (MINF) patients, and 30 cases of right ventricular abnormality (RVA) patients. Figure 4 shows the example cardiac MRI image along with its ground truth (GT) image. The dataset was separated into 100 training cases (with ground truth provided) and 50 testing cases (without ground truth provided). The ground truth is provided in both end-diastole (ED) and end-systole (ES) frames for reference segmentations of the right ventricle (RV) cavity, the myocardium (Myo), and the left ventricle (LV) cavity [4].

### 3.2. Experimental Setup

This section evaluates the segmentation performance of the proposed DeSPPNet model. The detailed evaluation protocols are as follows.

- A multiscale PPDM is integrated into the base network, with a variation of the following:
  - PPDM placement at the encoder layer 4, with feature map sizes of 28, 28, 48.
  - PPDM placement at the encoder layer 5, with feature map sizes of 14, 14, 640.
  - PPDM placement at the bottleneck layer (the deepest part of the network) with feature map sizes of 7, 7, 864.
  This placement position is the best position option as they have more complex features to allow the network to capture detailed features before upsampling and combining them with skip connections. The network benefits from rich, multiscale features during the encoding and decoding phases.
- For the purpose of alignment comparison, the PPDM will utilize pooling kernel sizes of 1 × 1, 2 × 2, 3 × 3, and 4 × 4, respectively. The coarsest pyramid level (1 × 1) will resemble global pooling, covering the entire image with a step size of 1. The finest level (4 × 4) will be set in the bottleneck level of the SPP module. Pooling kernel sizes above 4 will have the same feature size as 4 × 4, ensuring a maximum variety of 2, 3, and 4 parallel pathways. The use of variable bins in the described PPDM, with pooling kernel sizes of 1 × 1, 2 × 2, 3 × 3, and 4 × 4, is designed to produce a comprehensive multiscale representation by capturing features at different levels of granularity. By strategically combining these pooling levels, the module ensures maximum feature diversity while maintaining computational efficiency, allowing the multiscale representation to enhance segmentation performance across varying feature map resolutions.
- Additionally, this study conducted a comparison using an alternative multiscale module model, including the following:
  - ASPP, with the atrous convolution parameters (1, 2, 3, and 4) as dilation rates, and the maximum variety of 2, 3, and 4 parallel pathways.
  - Waterfall SPP, which is the combined SPP module in a cascade of convolution in a waterfall configuration.
  - Waterfall ASPP (WASPP), which is the combined ASPP in a cascade of convolution in a waterfall configuration.
- Finally, in this study, a benchmarking comparison will also be conducted between the proposed DeSPPNet and several existing CNN architectures: FC-DenseNet [28], TernausNet [29], U-Net [30], FCN-8 [31], and SegNet [32].

### 3.3. Performance Metrics

This work used three evaluation metrics to assess the segmentation performance of cardiac MRI, which are dice coefficient (dice), intersection over union (IoU), and accuracy (Acc). In multi-class segmentation problems, if A and B represent the sets of pixels for class c in the ground truth and predicted mask, respectively, and |S| denotes the number of elements in set S, the respective metrics can be computed as follows.

▪ Dice coefficient (dice): A measure of the similarity of two sets, usually used in semantic partitioning problems. As shown in Equation (2), the set is derived from the heart image segmented by the network structure and assesses the similarity between the two segmentation outputs and the reference image.
  (1) $$Dice\left(A,B\right)=2\frac{\left|A\cap B\right|}{\left|A\right|+\left|B\right|}$$
  Dice values range from 0 to 1, which denotes the entire spatial similarity between two datasets from binary segmentation, indicating total spatial overlap. A dice value close to 1 indicates that the heart image has been segmented accurately, with a high degree of overlap with the cardiac MRI image. Conversely, a dice value close to 0 suggests poor segmentation, with minimal overlap with the cardiac MRI image.
▪ Intersection over union (IoU): A measure of the overlap between the predicted segmentation and the ground truth.
  (2) $$IoU\left(A,B\right)=\frac{\left|A\cap B\right|}{\left|A\cap B\right|}$$
  It reflects how much of the predicted region corresponds to the actual region, and it penalizes both over-segmentation (including too many pixels in *A*) and under-segmentation (missing pixels in *B*).
▪ Accuracy (*Acc*): A proportion of correctly classified pixels to the total number of pixels in the image.
  (3) $$Acc\left(A,B\right)=\frac{\left|A\cap B\right|+\left|A^{c}\cap B^{c}\right|}{\left|A\cup B\right|}$$
  where $\left|A\cap B\right|$ is the number of pixels that are correctly predicted as belonging to the target class, $\left|A^{c}\cap B^{c}\right|$ is the number of pixels that are correctly predicted as not belonging to the target class, and $\left|A\cup B\right|$ is the total number of pixels (the union of ground truth and predicted sets).

## 4. Results and Discussion

### 4.1. Ablation Study of the DeSPPNet Performance

The initial learning rate (Lr) for our proposed network is set at 0.0001, using the Adam optimization algorithm for parameter updates. The dropout rate is fixed at 0.5 and batch normalization is employed as the regularization technique. The training batch size is configured to 4, and the model is trained for a maximum of 100 epochs to compensate for the effects of the reduced batch size. For labeling purposes, every pixel of the ground truth maps is normalized into 0–3 (where 0—background, 1—right ventricle (RV), 2—myocardium (Myo), and 3—left ventricle (LV)). Since the objective of the DeSPPNet development is to produce an accurate segmentation of all three cardiac chambers, the average value of all of the evaluation metrics will be used to determine the segmentation performance.

#### 4.1.1. Evaluation of the PPDM Placement at the Encoder Layer 4

The encoder layer of the DeSPPNet model plays a key role in progressively downsampling the input image while capturing detailed spatial and feature information. This layer has a feature map size of (28, 28, 448) that represents the following:

- 28 × 28: The spatial resolution of the feature map, indicating the height and width. This suggests that the input image has been downsampled to this size during the encoding process (through convolutions, pooling, or strides).
- 448: The number of feature channels or filters. This reflects the depth of the feature map and indicates the number of different feature detectors that have been applied.

This PPDM consists of nine DCUs and one SPP block. Each layer of DCU in the PPDM receives the feature maps of all preceding layers as input, and by the end of the block, SPP processes all the output feature maps from the last DCU to capture multiscale features. This multiscale dense connectivity pattern improves the gradient flow during training and allows the model to effectively reuse features and enable the network to gather both the fine-grained local features and the global context across multiple scales before downsampling in the encoder. The statistics of the automated segmentation performance result for the PPDM placement in encoder layer 4 are shown in Table 1. Upon closer inspection, the PPDM structure featuring four parallel paths (with a pooling kernel size of 1 × 1 and 4 × 4) yields the highest performance, with dice, IoU, and accuracy values of 0.852, 0.792, and 0.993, respectively.

**Figure 5:**
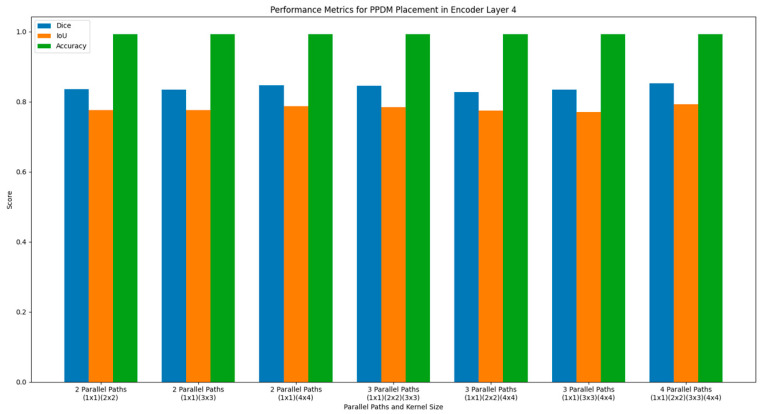
Performance metrics of DeSPPNet with PPDM applied at the encoder layer 4.

Figure 5 shows the bar chart to visualize the performance metrics for PPDM placement in encoder layer 4. The best result above suggests that at this stage of feature extraction process (a middle level of the network), the multiscale feature from four parallel paths produces the best segmentation performance. This PPDM structure effectively captures increased spatial details at this resolution through its four parallel paths. The improved segmentation performance is achieved by allowing richer multiscale contextual information while preserving spatial information at this level.

#### 4.1.2. Evaluation of the PPDM Placement at Encoder Layer 5

**Figure 6:**
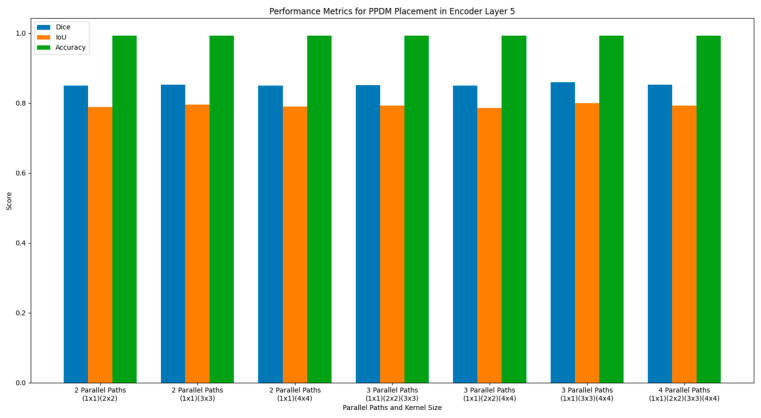
Performance metrics of DeSPPNet with PPDM applied at encoder layer 5.

The PPDM applied in encoder layer 5 consists of 11 dense blocks and a single SPP block. The feature map size for this placement configuration is (14, 14, 660). This layer’s lower spatial resolution significantly reduces the size of the feature maps. Pooling at different scales in the SPP block at the end of encoder layer 5 would now focus on capturing a more global context and improving contextual understanding rather than spatial precision. The statistics of the automated segmentation performance result for the PPDM placement in encoder layer 5 are shown in Table 2. The bar chart in Figure 6 shows that the three-parallel-path PPDM works best, with a pooling kernel size of (1 × 1), (3 × 3), and (4 × 4). This is shown by its 0.993 accuracy, 0.859 dice, and 0.800 IoU values. The performance results shown for this model also represent the best performance values among all variants that were proposed in this work.

At this deeper layer, the feature map size is smaller, and the number of feature channels (660) is higher. With three parallel paths, the model can capture enough multiscale information without unnecessary complexity or overfitting at this depth. Reducing to three paths could be effective because the smaller spatial dimension (14 × 14) may not require as many parallel paths to capture meaningful context, given the increased feature richness at this level.

#### 4.1.3. Evaluation of the PPDM Placement at the Bottleneck Layer

The bottleneck layer sits between the encoder and decoder and serves as the most abstract representation of the input. The bottleneck layer in DeSPPNet has a feature map size of (7, 7, 864), which means a very low spatial resolution of 7 × 7 and a high number of feature channels of 864, as it aggregates the feature representations from all preceding layers. The features in the bottleneck represent the most abstract, high-level information about the input, capturing complex patterns, objects, or relationships in the cardiac MRI image.

**Figure 7:**
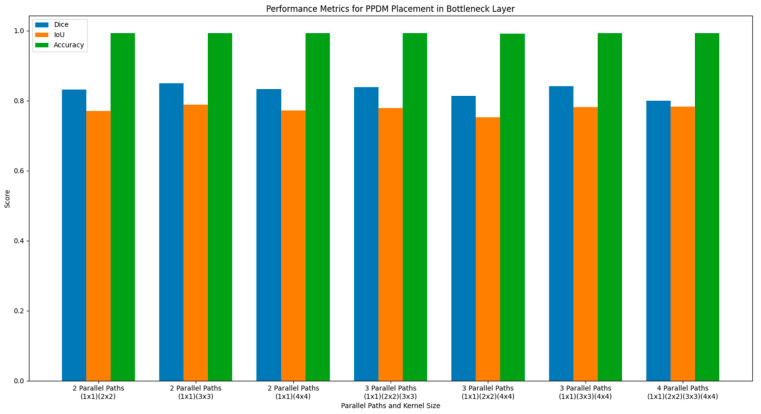
Performance metrics of DeSPPNet with PPDM applied at the bottleneck layers.

The PPDM applied in the bottleneck layer consists of 14 DCUs and a single SPP block. The decoder will now access a more comprehensive set of features that include global context and multiscale information derived from pooling operations of SPP blocks. The model performance table in Table 3 and performance metric bar chart in Figure 7 show that the two-parallel-path PPDM configuration (with pooling kernel sizes of (1 × 1), (2 × 2), (3 × 3), and (4 × 4)) works the best. This is shown by accuracy, dice, and IoU values of 0.993, 0.850, and 0.790, respectively. In the bottleneck layer, where the spatial resolution is further reduced, the PPDM configuration with only two parallel paths produces the best results. At this feature extraction stage, feature maps are even smaller in size but contain the most critical information about the image. Fewer parallel paths in this layer minimize spatial feature aggregation, preserving essential details without introducing excessive computational burden. This suggests that the PPDM benefits from fewer scales at this level, where the representation primarily encodes high-level, abstract features rather than detailed spatial information.

Different PPDM configurations benefit different depths in the encoder, highlighting an important point. Shallower layers, like encoder layer 4, keep more spatial information and need more parallel paths to capture multiscale details. Deeper layers, on the other hand, have more features but lower spatial resolution, so fewer parallel paths are needed to provide enough multiscale context. This strategy maximizes the segmentation accuracy by balancing trade-offs between multiscale detail capture and spatial feature compression at different depths. Across all PPDM configurations, the smallest pooling kernel size of (4 × 4) consistently produced the best results. This likely indicates that, regardless of the number of parallel paths or feature map resolution, fine-grained pooling retains essential information for cardiac MRI segmentation. Using a smaller pooling kernel can better capture subtle anatomical features critical for accurate segmentation in medical imaging.

The rationale for using variable bin sizes in PPDMs is rooted in their ability to address the inherent trade-offs between capturing multiscale details and preserving spatial resolution at different depths of the encoder. As the results suggest, different depths in the encoder benefit from tailored PPDM configurations that balance these trade-offs effectively.

In shallower encoder layers, feature maps retain more spatial resolution but contain fewer high-level semantic features. Applying fewer parallel paths with variable bin sizes in these layers should ensure the capture of both localized details and broader spatial context. This is crucial for cardiac MRI segmentation, where small anatomical features, such as thin myocardial walls, need to be identified alongside larger structures like the right ventricle and left ventricle. Variable bin sizes ensure that fine-grained features are preserved while also integrating contextual information from larger regions of the feature map.

In deeper encoder layers, where feature maps have more abstracted semantic information but lower spatial resolution, more parallel paths are needed. Variable bin sizes still play a critical role here by adapting to the reduced spatial information, ensuring that multiscale context is efficiently captured without redundant computation. By leveraging variable bin sizes, the PPDM dynamically adapts to the characteristics of feature maps at different encoder depths. This adaptability improves the model’s ability to integrate multiscale representations, leading to higher segmentation accuracy and robustness in handling the complex anatomical variability present in cardiac MRI.

**Figure 8:**
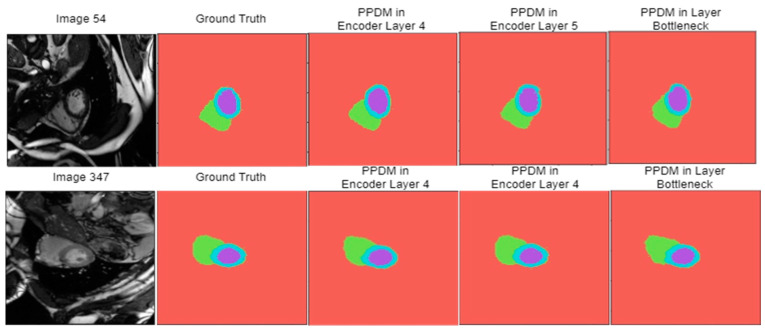
Some of the prediction images and their respective original images and ground truth maps, generated using DeSPPNet with PPDM applied at the encoder layers of 4, 5, and bottleneck.

Figure 8 shows the results of visual segmentation when the PPDM is placed at a certain layer of DeSPPNet’s encoder path. Based on the visual analysis, PPDM placement in encoder layer 5 consistently gave the best visual results in both prediction images. This method manages to capture the shape and size of the heart more closely. The cardiac MRI visual result from the PPDM configuration at encoder layer 4 suggests a slight underestimation of the heart’s size, specifically the left ventricle, in image 54, and a slight overestimation of the left ventricle and overestimation of the right ventricle in image 347. In both images, the visual segmentation result of the PPDM configuration in the bottleneck layer is reasonably accurate, although there are some minor deviations, especially in the region between the ventricles. The visual analysis concludes that applying SPP at layer 5 produces the most accurate segmentations for both images.

### 4.2. Performance Comparison with Other Multiscale Structures

This section will benchmark the performance of several multiscale structures, including SPP, ASPP, Waterfall SPP, and Waterfall ASPP. The basis for DeSPPNet is built upon the SPP module, and other multiscale modules such as ASPP will be assessed for benchmarking purposes. The “Waterfall” variants of the multiscale modules, namely, Waterfall SPP and Waterfall ASPP, are also introduced. 

The implementation of the four multiscale modules will be ensured to produce optimal module placement, as discussed in the previous section, specifically on encoder layer 5. As shown in Table 4, the proposed PPDM configuration still provides the best performance, with accuracy, dice, and IoU values of 0.993, 0.859, and 0.800, respectively. This PPDM configuration enables the DeSPPNet to capture robust spatial information and broad contextual information without compromising detailed resolution, allowing for the accurate delineation of cardiac anatomical structures with large variations, from the fine details of the myocardium to the large structures of the ventricles.

The PPDM configuration using the multiscale structure of ASPP performed the second best, with accuracy, dice, and IoU values of 0.992, 0.849, and 0.791, respectively. While atrous convolutions excel at capturing a bigger global context, some critical aspects of cardiac MRI segmentation have been less effectively preserved, especially for fine details of the object boundaries. The dilation factor may lead to slight precision loss at the edges, resulting in slightly lower performance compared to DeSPPNet, which is better at retaining these details through SPP’s multiscale pooling.

The waterfall method did not yield superior results for either the multiscale SPP or ASPP techniques. The accuracy, dice, and IoU values of the waterfall SPP were 0.992, 0.847, and 0.788, respectively, while the waterfall ASPP yielded values of 0.992, 0.842, and 0.783, respectively. The waterfall method might simplify the feature fusion process by facilitating the combination of multiscale features, but this process reduces segmentation accuracy by potentially losing the finer details needed for high-precision segmentation, such as the thin lines between heart structures in cardiac MRI images.

**Figure 9:**
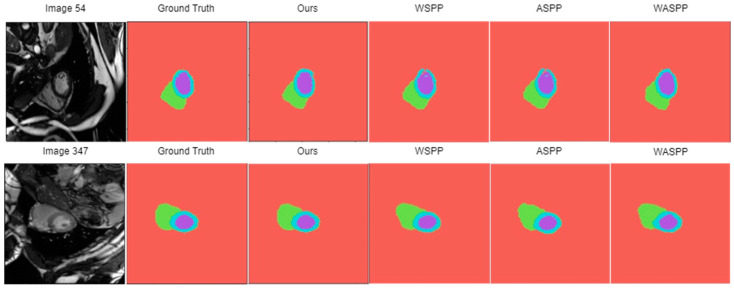
Some of the prediction images with their respective original images and ground truth maps, tested using different multiscale structures (SPP, WSPP, ASPP, and WASPP) applied at encoder layer 5.

Figure 9 illustrates the visual segmentation outcomes of several multiscale structures used in the PPDM configuration. Based on the visual analysis, SPP and ASPP consistently outperform WSPP and WASPP in terms of segmentation accuracy. In both images’ segmentation results, SPP provides the most accurate segmentation, with minimal deviations from the ground truth. The ASPP segmentation is reasonably accurate, although there are some minor deviations, especially in the region between the ventricles. The WSPP segmentation slightly underestimates the size of the left ventricle and overestimates the size of the right ventricle. The WASPP tends to underestimate the size of objects, particularly in images with complex structures.

### 4.3. Performance Comparison with Other CNNs

In the context of cardiac MRI segmentation, achieving high accuracy in distinguishing cardiac structures is essential for supporting clinical diagnosis and treatment planning. Developing and validating a novel network architecture, such as the proposed DeSPPNet, involves more than just examining its standalone performance. A robust evaluation requires a systematic comparison with other CNN-based segmentation networks. This comparative analysis helps highlight the strengths and potential limitations of the proposed model while ensuring that the observed improvements are due to architectural innovations rather than variations in training configurations.

All benchmarked models have been tested with a set of optimized hyperparameters for a fair comparison. This standardized setup enables a direct and unbiased comparison of the proposed DeSPPNet with other networks like FC-DenseNet, TernausNet, U-Net, FCN-8, and SegNet. By benchmarking SPPDenseNet against a diverse set of architectures, a better understanding is gained of how the inclusion of multiscale dense connections in SPPDenseNet affects its ability to capture complex spatial details and contextual information in CMRI images. Additionally, this comparison highlights which architectural features such as dense connections, skip connections, and multiscale pooling are the most effective in addressing the specific challenges of cardiac MRI segmentation.

As shown in Table 5, the proposed DeSPPNet outperforms other networks due to its efficient integration of multiscale pooling and dense connectivity, capturing both detailed and contextual information effectively. FC-DenseNet, while lacking explicit multiscale pooling, ranks relatively highly due to its dense connections that enhance feature reuse and spatial detail retention. Networks like TernausNet and U-Net perform well due to their skip connections or pre-trained backbones, but lack the multiscale and dense structures that can enhance fine detail handling in complex cardiac MRI segmentation. On the other hand, architecture like SegNet uses a standard encoder–decoder network, but it lacks skip connections to support the decoder part. This result shows reduced detail retention compared to U-Net or FC-DenseNet. Without skip connections, the network struggles to reconstruct precise boundary details, leading to lower segmentation accuracy, particularly for cardiac MRI. This analysis highlights the importance of multiscale and dense connections in deep network design for cardiac MRI segmentation, where both local and global anatomical details must be precisely delineated.

**Figure 10:**
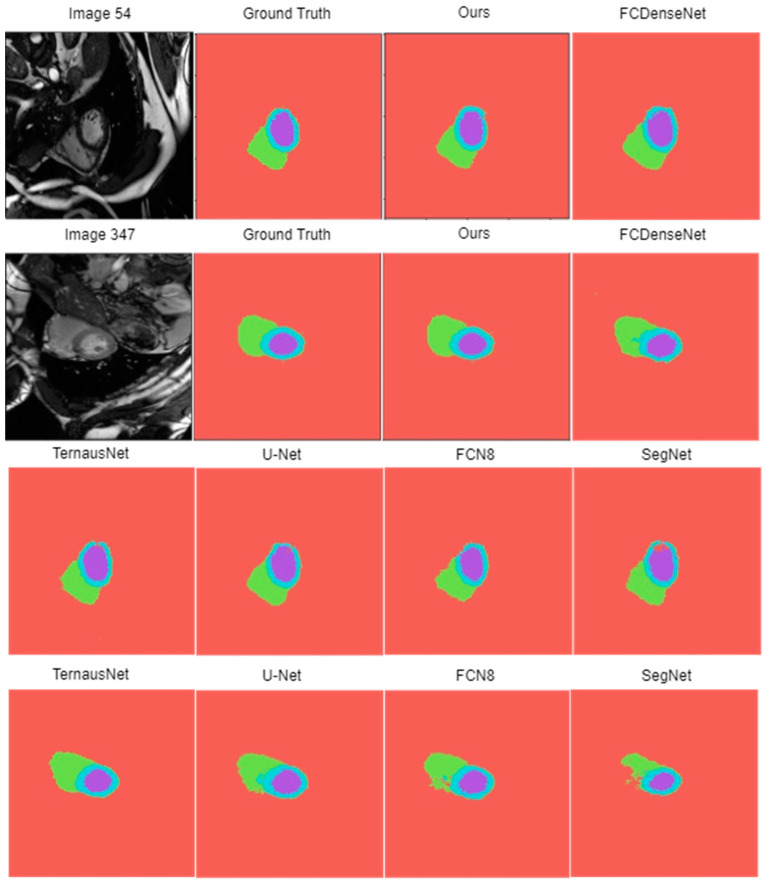
Segmentation results of the proposed network and other benchmarked CNNs.

Based on the visual analysis of Figure 10, the proposed method produces the most accurate segmentation maps, closely matching the ground truth maps. FC-DenseNet also performs reasonably well, but this network struggles in cases with significant variations in sizes and scales. TernausNet, U-Net, and FCN8 struggle to accurately segment the complex cardiac structures, leading to significant deviations from the ground truth. SegNet exhibits the poorest performance due to its difficulty in accurately segmenting cardiac structures. This is likely due to a loss of fine-grained details, which is crucial for the accurate boundary delineation of each cardiac chamber.

## 5. Conclusions

In this work, the optimal configuration of SPP paths is devised, which is highly dependent on the feature size and layer depth within the encoder to produce the best cardiac segmentation maps. In general, earlier encoder layers (with higher resolutions) benefit from having more parallel paths in the SPP, which provide a richer multiscale representation in capturing fine-grained details. Conversely, with a deeper layer placement of the SPP in the encoder, where features are more compressed, the model consists of fewer paths to achieve an effective balance between multiscale context and computational efficiency. Hence, this adaptive use of SPP paths across layers contributes significantly to achieving high segmentation performance with the cardiac MRI data. The developed multiscale deep learning model produces high-quality segmented images, which enhances organ visualization that helps clinicians to better communicate the diagnosis results to patients. The algorithm also reduces subjective biases, ensuring consistent diagnosis results across different cases and clinicians. Furthermore, automating the segmentation process will significantly reduce the time required for the manual delineation of cardiac structures, enabling quicker diagnosis and treatment planning. Finally, the proposed automated segmentation can be integrated with clinical workflows, which allows clinicians to focus on interpretation tasks rather than performing the time-consuming manual segmentation of organ structures.

## Figures and Tables

**Table 1:** Performance evaluation for PPDM placement in encoder layer 4.

PPDM Placement	Number of Parallel Paths	Kernel Size	Performance Metrics		
			Dice	IoU	Acc
Encoder Layer 4	2	(1 × 1) (2 × 2)	0.836	0.776	0.992
		(1 × 1) (3 × 3)	0.835	0.776	0.992
		(1 × 1) (4 × 4)	0.847	0.787	0.992
	3	(1 × 1) (2 × 2) (3 × 3)	0.845	0.784	0.992
		(1 × 1) (2 × 2) (4 × 4)	0.827	0.775	0.992
		(1 × 1) (3 × 3) (4 × 4)	0.834	0.77	0.992
	4	(1 × 1) (2 × 2) (3 × 3) (4 × 4)	0.852	0.792	0.993

**Table 2:** Performance evaluation for PPDM placement in encoder layer 5.

PPDM Placement	Number of Parallel Paths	Kernel Size	Performance Metrics		
			Dice	IoU	Acc
Encoder Layer 5	2	(1 × 1) (2 × 2)	0.849	0.789	0.992
		(1 × 1) (3 × 3)	0.853	0.795	0.992
		(1 × 1) (4 × 4)	0.85	0.79	0.993
	3	(1 × 1) (2 × 2) (3 × 3)	0.851	0.793	0.992
		(1 × 1) (2 × 2) (4 × 4)	0.849	0.786	0.993
		(1 × 1) (3 × 3) (4 × 4)	0.859	0.8	0.993
	4	(1 × 1) (2 × 2) (3 × 3) (4 × 4)	0.852	0.793	0.992

**Table 3:** Performance evaluation for PPDM placement in bottleneck layer.

PPDM Placement	Number of Parallel Paths	Kernel Size	Performance Metrics		
			Dice	IoU	Acc
Bottleneck Layer	2	(1 × 1) (2 × 2)	0.832	0.771	0.992
		(1 × 1) (3 × 3)	0.849	0.789	0.993
		(1 × 1) (4 × 4)	0.833	0.772	0.993
	3	(1 × 1) (2 × 2) (3 × 3)	0.839	0.779	0.992
		(1 × 1) (2 × 2) (4 × 4)	0.814	0.753	0.991
		(1 × 1) (3 × 3) (4 × 4)	0.841	0.781	0.992
	4	(1 × 1) (2 × 2) (3 × 3) (4 × 4)	0.8	0.783	0.992

**Table 4:** Result comparison of the proposed network with PPDM using different multiscale structures.

PPDM Placement	Type of Multiscale Structure	Performance Metrics		
		Dice	IoU	Acc
Encoder Layer 5	DeSPPNet	0.832	0.771	0.992
	ASPP [33]	0.849	0.789	0.993
	WSPP [34]	0.833	0.772	0.993
	WASPP [35]	0.839	0.779	0.992

**Table 5:** Performance comparison of the DeSPPNet with other CNNs.

Model	Performance Metrics		
	Dice	IoU	Acc
DeSPPNet	0.832	0.771	0.992
FC-DenseNet [28]	0.641	0.591	0.975
TernausNet [29]	0.639	0.582	0.970
U-Net [30]	0.632	0.576	0.968
FCN8 [31]	0.589	0.547	0.978
SegNet [32]	0.545	0.547	0.983

## Data Availability

The data presented in this study are available at https://www.creatis.insa-lyon.fr/Challenge/acdc/databases.html (accessed on 5 March 2024).
